# Community Interventions to Improve Glycemic Control in African Americans With Type 2 Diabetes: A Systemic Review

**DOI:** 10.5539/gjhs.v7n5p171

**Published:** 2015-02-24

**Authors:** Brittany L. Smalls, Rebekah J. Walker, Heather S. Bonilha, Jennifer A. Campbell, Leonard E. Egede

**Affiliations:** 1 Center for Health Disparities Research, Medical University of South Carolina, Charleston, SC, USA; 2 Department of Health Science & Research, College of Health Professions, Medical University of South Carolina, Charleston, SC, USA; 3 Department of Medicine, Division of General Internal Medicine and Geriatrics, Medical University of South Carolina, Charleston, SC, USA; 4 Health Equity and Rural Outreach Innovation Center, Charleston VA COIN, Ralph H. Johnson VA Medical Center, Charleston, SC, USA

**Keywords:** type 2 diabetes, systematic review, community interventions, glycemic control

## Abstract

**Purpose:**

The purpose of this study was to conduct a systematic review of published community interventions to evaluate different components of community interventions and their ability to positively impact glycemic control in African Americans with T2DM.

**Methods:**

Medline, PsychInfo, and CINAHL were searched for potentially eligible studies published from January 2000 through January 2012. The following inclusion criteria were established for publications: (1) describe a community intervention, not prevention; (2) specifically indicate, in data analysis and results, the impact of the community intervention on African American adults, 18 years and older; (3) measure glycemic control (HbA1C) as an outcome measure; and (4) involve patients in a community setting, which excludes hospitals and hospital clinics.

**Results:**

Thirteen studies out of 9,233 articles identified in the search met the predetermined inclusion criteria. There were 5 randomized control trials and 3 reported improved glycemic control in the intervention group compared to the control group at the completion of the study. Of the 8 studies that were not randomized control trials, 6 showed a statistically significant change in HbA1C.

**Conclusion:**

In general, the community interventions assessed led to significant reductions in HbA1C in African Americans with type 2 diabetes. Community health workers did not have a greater impact on glycemic control in this sample. The findings of this study provides insight for designing community-based interventions in the future, such as including use of multiple delivery methods, consideration of mobile device software, nutritionist educator, and curriculum-based approaches.

## 1. Introduction

### 1.1 Burden of Diabetes

Type 2 diabetes mellitus (T2DM) affects more than 25.3 million people in the United States (US) (National Institute for Diabetes, Digestion, and Kidney Disease [NIDDK], 2011). It is predicted, by 2050, there will be 29 million individuals with T2DM in the US (Narayan, Boyle, Thompson, Sorenson, & Williamson, 2003). In 2007, the overall estimated cost of T2DM in the US, including direct and indirect costs, equaled $174 billion and the cost is expected to reach $192 billion by 2020 (NIDDK, 2011). Major complications and comorbid illnesses result from T2DM, including blindness and vision problems, nervous system disorders, kidney disease, amputations, periodontal disease, heart disease, and stroke (NIDDK, 2011). In addition, T2DM is the seventh leading cause of death based on US death certificates in 2007 (NIDDK, 2011).

### 1.2 Burden of Diabetes on African Americans

Minority populations, particularly African Americans, are disproportionately affected by T2DM (Narayan et al., 2003). African Americans are 1.6-times more likely to develop T2DM and suffer from complications compared to non-Hispanic Whites (Narayan et al., 2003). The average years of life lost for African American males due to T2DM is 9.3 years in contrast to 8 years in non-Hispanic White males (Narayan et al., 2003). Similarly, the average number of years lost for females due to T2DM is 12 years in African Americans compared to 10.3 years in non-Hispanic Whites (Narayan et al., 2003). These statistics become even more pertinent based on projections that T2DM will increase 3.0-fold in African Americans, and 1.2-fold in non-Hispanic Whites by 2020 (Hogan, Dall, & Nikolov, 2003).

Evidence shows that minority populations have higher mortality rates due to complications associated with T2DM (Campbell, Walker, Smalls, & Egede, 2012). Poor self-management behaviors and poor T2DM clinical outcomes are suggested as the reasons for higher mortality and complication rates. Based on the American Diabetes Association (ADA) guidelines for glycemic control, blood pressure and lipids, on average, African Americans have suboptimal T2DM-related outcomes and have an increased risk for microvascular and macrovascular complications (Campbell et al., 2012). For the purposes of this review will focus on glycemic control, as the primary outcome, measured by hemoglobin A1C (HbA1C), which is a known indicator of T2DM severity (ADA, 2013).

### 1.3 Barriers to Optimal Health Outcomes in African Americans

Traditional approaches to T2DM management, based on studies in primarily non-Hispanic White populations, may not be as effective in African Americans due to differing social determinants. Disparities in optimal T2DM management have been attributed to barriers at the patient-, provider-, and health systems-levels, although many of these barriers have not been adequately studied (Syler & Oddo, 2002; Marmot, 2005; Brown et al., 2004). At the patient-level, three important barriers to optimal outcomes have been identified: lack of T2DM specific knowledge, poor self-management skills, and poor motivation to make lifestyle behavior changes (Syler & Oddo, 2002). These barriers are thought to explain the lower likelihood of African Americans adhering to a healthy diet, engaging in regular physical activity, and participating in weight loss programs (Tang et al., 2005). Provider- and health system-level barriers to proper T2DM care include lower rates of screening, perceived complexity and difficulty of treating patients and lack of adequate time and resources (Narayan et al., 2003; Tang et al., 2005). Although several novel interventions are available to manage T2DM, minority groups are less likely to attain target glycemic levels set by the American Diabetes Association (Syler & Oddo, 2002).

### 1.4 Community Interventions and Diabetes Self-Care

Efforts to implement interventions targeting African American with T2DM in a health care setting have shown to reduce HbA1C by 0.8% (Ricci-Cabello et al., 2013). Yet, in recent years, health interventions implemented in nonclinical settings has increased, allowing for novel patient focused study components as can be seen in community interventions. One way this multi-level problem has been addressed is through community interventions (Ricci-Cabello et al., 2013).

For the purpose of this review, community interventions are techniques that introduce and promote diabetes self-care management outside of the hospital or hospital clinics. These interventions may be more effective than traditional methods because they address sociocultural and environmental factors that positively influence T2DM health outcomes (Nine, Lakies, Jarrett, & Davis, 2003; Norris et al., 2002; Englegau et al., 1998; Liburd, Jack, Williams, & Tucker, 2005; Jack, Liburd, Vinicor, Brody, & Murry, 1999; Brody, Jack, Murry, Landers-Potts, & Liburd, 2001; Egan, Tannahill, Petticrew, & Thomas, 2008; Hausmann, Ren, & Sevick, 2010). It has been suggested that systematically addressing socioeconomic and environmental factors can attenuate observed minority disparities in T2DM health outcomes, and may address some of the barriers to optimal outcomes (Fry, Gleeson, & Rissel, 2010; Maddigan, Feeny, Majumdar, Farris, & Johnson, 2006; McKinlay & Marceau, 2000; Bierman & Dunn, 2006; Whiting, Unwin, & Roglic, 2010).

Community interventions have been proposed as a better way to reach African Americans due to the information presented and the approach used. Evidence-based information is presented in a culturally competent manner and specific components of and resources for an individual’s T2DM are provided (Englegau et al., 1998). This approach has the ability to address specific challenges and ways to overcome poor T2DM self-management (Englegau et al., 1998). Additionally, the approach is more comprehensive and usually tailored to accommodate the intended participants by focusing on reducing risk factors, prevention of complications and disabilities, and improving quality of care (Englegau et al., 1998).

The use of community health workers in community interventions has been shown to improve health behaviors and outcomes (Spencer et al., 2011). Community health workers are characterized as members of the target community who have been trained to be liaisons between their communities and healthcare providers (Spencer et al., 2011). Their duties include educating patients, identifying resources, and becoming part of the patients’ social support network (Quinn et al., 2008). Community health workers seem to have a positive influence in racial and ethnic minority groups who have historically had restricted access to health care services (Spencer et al., 2011).

### 1.5 Systematic Review Objective

Though, theoretically, community health workers may factor more importantly in the success of community interventions than other elements of community interventions, this comparison has not been investigated. This systematic review was conducted in an effort to evaluatevariouscommunity interventions and their ability to positively impact glycemic control in African Americans with T2DM.

## 2. Methods

### 2.1 Search Strategy and Databases

A search for articles published from January 2000 through January 2012 using 3 databases, Medline, PsychINFO, and CINAHL, was conducted for potentially eligible studies using a reproducible strategy. The search was limited to 12 years because the majority of interventions targeting African Americans with T2DM were published starting in 2000 (Ricci-Cabello et al., 2013). The following separate searches were conducted using medical subject heading (MeSH) terms to maximize the search results. MeSH terms, as defined by the Cochrane Library (Cochrane), are a set of terms naming descriptors in hierarchical structure that allows one to search at various levels of specificity. The first search used the search terms diabetes and ethnic groups. The second search used the terms diabetes and lifestyle. The third search used the terms diabetes and community. The three separate searches, combined, resulted in 9,233 citations. Duplicates were removed.

### 2.2 Selection Criteria

**Figure 1:**
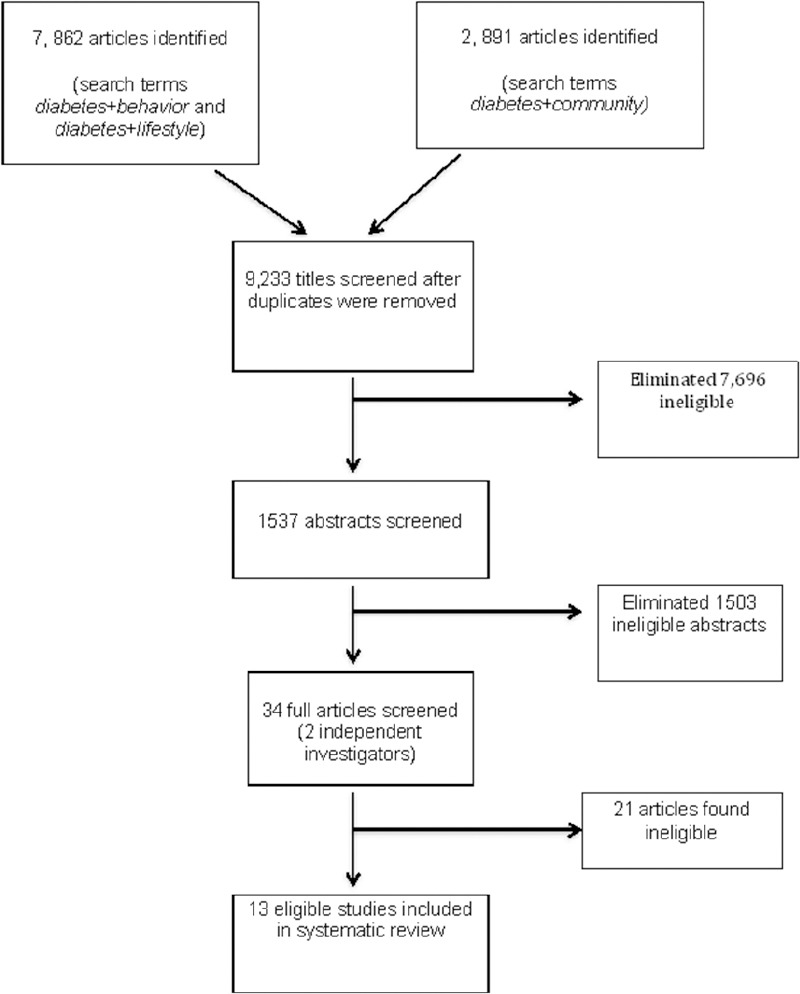
Eligible article selection process

The following inclusion criteria were implemented: (1) describe an intervention whose aim is to improve health outcomes, not prevention of T_2_DM; (2) specifically indicate in data analysis and results the impact of the community intervention on African American adults, 18 years and older; (3) indicate HbA1C as an outcome measure to determine successful diabetes management; (4) involve patients in a community setting which excludes hospitals and hospital clinics; and (5) articles must be published, no grey material were included. No articles were excluded based on study design. However, articles were excluded if titles indicated that the study was focused on gestational diabetes or type 1 diabetes. Full articles were read and reviewed by 2 independent researchers (BS, RW) using a study specific checklist that outlined the inclusion and exclusion criteria to assist with consistency between reviewers (Ueffing, Tugwell, Welch, Petticrew, & Kristjannson, 2011). In the case of a disagreement regarding article inclusion, a senior investigator (LE) was asked to make the final decision. Thirteen eligible studies were identified based upon the predetermined inclusion criteria (Figure 1).

### 2.3 Data Collection

Data was obtained on the number of study participants; race/ethnicity of the sample population; duration of the intervention; setting of the intervention; and intervention description including the community intervention being implemented, theoretical basis for study, study design, type of control, and outcome (HbA1C) (Table 1). Each article was reviewed for statistical significance of the outcome variable, HbA1C. Mean baseline of A1C, community intervention group mean change in HbA1C, control group mean change in A1C, and statistical significance (p<0.05) were documented (Table 2). The target threshold for glycemic control was an HbA1C less than 7%, based on ADA guidelines (Hogan et al., 2003). Finally, each article was reviewed for the use of community health workers, in addition to other relevant characteristics of the intervention, including use of a culturally tailored approach, curriculum-based approach, involvement of nurse educators, diabetes educators, nutritionist educators, one-on-one counseling, group counseling, physician involvement, supervised exercised, telemedicine, nurse case managers, and/or mobile device software (Table 3). No quantitative data synthesis was performed because of the heterogeneous interventions across studies and diversity of the study designs and outcome measures.

**Table 1:** Summary of studies meeting inclusion criteria

Characteristics	Research Studies				
Study Author, Year, Study Design	Sekhobo et al., 2008, Retrospective	Gary et al., 2009, RCT	Melkus et al., 2004, Pretest-posttest one group	Hargraves et al., 2012, RCT
Number of Participants (No. completed)	138 (132)	186 (149)	25 (25)	1415 (1415)
Race/Ethnicity	AA, NHW, HW	AA	AA	NHW, HW, AA
Duration of Intervention	22 months (mean interval between 1^st^ and 4^th^ study visits)	24 months	6 weeks	24 months
Setting of Intervention	New York City, NY community clinics	East Baltimore, MD community clinics	Urban community general clinical research center	Community health center
Intervention Description	4-visits w/a NCM for diabetes education and self-management	Randomized into NCM+UMC, CHW+UMC or UMC+NCM+CHW3 visits/yr of 45-60 minsessions with NCM and/or CHW	Written materials and videotapes of AAs promoting diabetes management	16, 3-hr modules for CHWs and 6 hrs of training for supervisors
Theoretical Basis	NR	Precede-proceed	Social Learning Theory and Transtheoretical Model of Behavior Change	Ecological framework
Type of Control	Clinic that did not have a NCM	UMC	NR	6 CHCs without CHWs
Limitations	-Selection bias -Variability in data collection -Unable to isolate effects of NCM	-No. of potential participant was small -Volunteer bias -Variable time of participant follow up	-Volunteer bias -Small sample size -Two-group design	-Rresources were provided by the state government (not generalizable to other states) -Weak matching design -Minimal contact by CHW

Study Author, Year, Study Design	Nine et al., 2003, Quasi-experimental	Two Feathers et al., 2005, Nonrandomized 1-group before and after	Utz et al., 2008, Quasi-experimental	Quinn et al., 2011, Clustered-randomized clinical trial
Number of Participants (No. completed)	75 (44)	151 (111)	22 (21)	213 (163)
Race/Ethnicity	AA	AA, Latino	AA	AA, NHW
Duration of Intervention	12 months	5 months	8 weeks	12 months
Setting of Intervention	Fairfield community of West Virginia	Detriot, MI	Community center, central Virginia	Community primary care setting
Theoretical Basis	Cultural competence	Cultural competence; Empowerment	Social cognitive theory	NR
Type of Control	NR	Usual care	NR	UMC
Limitations	-At the end of 1 yr of intervention the researchers could not measure the effect of each outcome on each intervention component -Variable enrollment times	-Difficult to draw conclusions because non-experimental design may contribute to selection bias	-Small sample size -Brief 10 week follow up	-mixed-effects model analysis used which imputes missing data -WEE analysis was used to increase the weight of data that were similar to participants with missing data

Study Author, Year, Study Design	Murrock et al., 2009, Mixed-methods design	Speer et al., 2008, Pretest/posttest design	Quinn et al., 2008, Non blinded randomized control trial	Spencer et al., 2011, Randomized delayed control group
Number of Participants (No. completed)	46 (46)	351 (144)	30 (26)	183 (136)
Race/Ethnicity	AA	AA, NHW	AA, NHW	AA, Latino
Duration of Intervention	12 weeks	4 months	3 months	6 months
Setting of Intervention	Community-based outpatient clinic, Midwest	Georgia, senior citizens centers	Maryland, community clinics	Detroit, MI; community centers
Intervention Description	12 weeks of dance, 2 sessions per week; focus groups	8, 40-60 min sessions; pre-intervention test, HbA1c measurement; post-intervention test, HbA1c measurement	Utilization of WellDoc System software to help monitor patient HbA1c	8, 2-hour sessions with a CHW every 2 weeks
Theoretical Basis	Social cognitive theory with complexity theory	Health belief model	NR	Empowerment; motivational Iiterature
Type of Control	UMC	NR	UMC	UMC
Limitations	-Small sample size -Lack of generalizability (volunteer bias)	-Concerns of effect of functional limitations on participation -No control group -Variability in implementation	-Limited generalizability -Self-reported improvements in self-management skills for UMC group	-Small sample size -Self-reported behavioral measures -Clinical measurement timelines varied

NR=not reported in the research article; NCM=nurse case manager; UMC=usual diabetes medical care; CHW=community health worker; AA=African American; NHW=non Hispanic white; HW=Hispanic white; PCP=primary care physician.

**Table 2:** Outcomes of studies meeting inclusion criteria

Characteristics	Studies				
Study author, year	Sekhobo et al., 2008	Gary et al., 2009	Melkus et al., 2004	Hargraves et al., 2012	Nine et al., 2003
Mean baseline HbA1c (%)	Good: 6.1± 0.7% Intermediate: 7.8±0.5% Poor: 11.0±1.6%	UMC=8.5%±2% NCM=8.8±2.2% CHW=8.4±2% NCM/CHW=8.6±1.9%	8.0%	8.0%	7.82%
Intervention description	4 visits with a NCM focusing on DSME	NCM, CHW, or CHW+NCM; 3 visits/yr 45-60 min session with NCM and/or CHW	Written materials and videotapes of African Americans promoting diabetes management	16, 3-hour modules for CHWs and 6 hours of training for facility supervisors	Chronic disease management program
Intervention mean change in HbA1c (%) and statistical significance	Visit1=-0.46%, p<0.05 Visit2=-0.89%, p<0.05 Visit3=-1.34%, p<0.05 Visit4=-0.9%, p<0.05	NCM=-0.3±0.49% CHW=-0.3±0.49% NCM+CHW=-0.8±0.52% *compared to UMC NCM and CHW was statistically significant for within group change from baseline, p<0.05	NR	3.8%, p>0.05	0.51%, p=0.105; however those whose mean HbA1c>7% there was a -1.29%, p<0.05
Control mean change in HbA1c (%) and statistical significance	NR	NR	NR	-0.3%, p>0.05	NR
Post intervention HbA1c (%)	NR	NR	3 month post intervention follow-up: 6.9 %, p=0.002	NR	NR

Study author, year	Two Feathers et al., 2008	Utz et al., 2008	Quinn et al., 2011	Magee et al, 2011	Murrock et al., 2009
Mean baseline HbA1c (%)	Intervention=8.4±2.3% Control=8.4±2.0%	NR	NR	NR	NR
Intervention description	5, 2-hour meetings every 2 weeks	8, 2-hour sessions of DSME to individuals or group	Diabetes software for mobile device (coach only); coach+PCP; PCP+coach+decision support	2, 2.5-hour session focused on specific aspects of the ABCs of Diabetes and Strategies on how to communicate with their physician	12 weeks of dance, 2 sessions per week; focus groups
Intervention mean change in HbA1c (%) and statistical significance	-0.8%, p<0.001	Individuals: 0.32%, p=0.855 Group:0.24%, p=0.111	-1.2%, p<0.001 compared to control group	-0.55%, p<0.001; the percentage of those who met HbA1c<7% increased significantly as well (p<0.001)	-0.5%, p<0.05
Control mean change in HbA1c (%) and statistical significance	-0.2%, p=0.160	NR	NR	NR	-0.3%, p<0.05
Post intervention HbA1c (%) and statistical significance	NR	NR	NR	NR	NR

Study author, year	Speer et al., 2008	Quinn et al., 2008	Spencer et al., 2004	Study author, year	Speer et al., 2008
Mean baseline HbA1C, %	7.0%	Intervention: 9.51% Control: 9.05%	Intervention:8.6% Control:8.5%	Mean baseline HbA1C, %	7.0%
Intervention description	8, 40-60 min session; pre-intervention test and HbA1c; post intervention test and HbA1c	Utilization of WellDoc system software to help monitor participants’ diabetes outcomes	8, 2-hour sessions with a CHW every 2 weeks	Intervention description	8, 40-60 min session; pre-intervention test and HbA1c; post intervention test and HbA1c
Intervention mean change in HbA1C, %	-0.25%, p=0.001	-2.03%, p=0.04	-0.8%	Intervention mean change in HbA1C, %	-0.25%, p=0.001
Control mean change in HbA1C, %	NR	-0.68%, p=0.04	No change	Control mean change in HbA1C, %	NR
Post intervention HbA1C, %	NR	NR	6-month post intervention follow-up -0.8%, p<0.01; intervention effect -9.7, p<0.01	Post intervention HbA1C, %	NR

NR=not reported in the research article.

**Table 3:** Types of interventions

Study Author, Year	Nurse Case Manager	Telemedicine	Culturally Tailored	Mobile Device Software	Nurse Educator	Nutritionist Educator	Diabetes Educator	Curriculum-based Intervention	One-on-one Counseling	Group Counseling	Physician Involvement	Supervised Exercise	Community Health Worker	Statistically Significant
Sekhobo et al, 2008	**X**													**X^a^**
Gary et al, 2009		**X**			**X**				**X**				**X**	
Melkus et al, 2004			**X**		**X**	**X**					**X**		**X**	**X^a^**
Hargraves et al, 2012									**X**				**X**	
Nine et al, 2003					**X**	**X**				**X**		**X**		**X^a^**
Two Feather et al, 2005								**X**		**X**			**X**	**X^a^**
Utz et al, 2008			**X**				**X**		**X**		**X**			
Quinn et al, 2011				**X**			**X**		**X**			**X**		**X^a^**
Murrock et al, 2009										**X**		**X**		**X^b^**
Speer et al, 2008				**X**		**X**	**X**	**X**				**X**		**X^a^**
Quinn et al, 2008				**X**						**X**	**X**			**X^a^**
Spencer et al, 2011									**X**	**X**			**X**	**X^b^**
Magee et al, 2011			**X**					**X**						**X^a^**

a Significant change between intervention groups,

b Significant change within the control group and intervention group.

## 3. Results

### 3.1 Study Selection

Figure 1 shows the search results. 9,233 citations resulted from the search after duplicates were removed. Reviewing titles produced 1537 abstracts, after which 34 articles were determined eligible for full review. Of the 34 full articles reviewed, 13 met the predetermined inclusion criteria.

### 3.2 Study Characteristics

The information collected on evidence and outcomes from eligible articles are shown in Tables 1 and 2. Study design varied greatly amongst these articles. Five were randomized control trials (Spencer et al., 2011; Gary et al., 2009; Hargraves, Ferguson, Lemay, & Pernice, 2012; Quinn et al., 2011; Quinn et al., 2008), 2 used pretest/posttest design (Melkus et al., 2004; Speer et al., 2008), 2 were quasi-experimental (Nine et al., 2003; Utz et al., 2008), one was retrospective (Sekhobo, Wang, & Ferrari, 2008), 1 was a randomized 1-group before and after study (Two Feathers et al., 2005), 1 was a cohort study (Magee et al., 2011), and 1 used a mixed methods design (Murrock, Higgins, & Killion, 2009). Sample sizes of the studies ranged from 22 to 1,415 participants and intervention duration ranged from 2 sessions (unspecified duration) to 24 months (Table 1). The average baseline glycemic control was poor (HbA1C>7%) in most of these studies and the average sample size for the reviewed studies was 246 participants.

### 3.3 Characteristics of Community Interventions

The community interventions utilized various delivery methods, such as software, telemedicine, and in-person sessions. Additionally, various types of healthcare professionals delivered the intervention information, including nurse case managers, community health workers, and diabetes educators. The type of information provided, if specified, was curriculum-based or culturally tailored. These community interventions were implemented via community clinics, community centers, home visits (i.e., nurse case managers or community health workers visiting the homes of individuals with T2DM to provide diabetes self-management education), or independent in-person interactions (i.e., mobile phone software that assists in monitoring HbA1C). The racial composition of study participants varied and included non-Hispanic Whites, Hispanic Whites, Latinos, and African Americans.

Intervention delivery methods were categorized for the 13 reviewed articles (Table 3). Categories included group counseling (Nine et al., 2003; Spencer et al., 2011; Quinn et al., 2008; Two Feathers et al., 2005; Murrock et al., 2009), use of community health worker (Spencer et al., 2011; Gary et al., 2009; Hargraves et al., 2012; Melkus et al., 2004; Two Feathers et al., 2005), one-on-one counseling (Spencer et al., 2011; Gary et al., 2009; Hargraves et al., 2012; Quinn et al., 2008; Utz et al., 2008), supervised exercise (Nine et al., 2003; Quinn et al., 2011; Speer et al., 2008; Murrock et al., 2009), use of culturally tailored approaches (Melkus et al., 2004; Utz et al., 2008; Magee et al., 2011), use of mobile application software (Quinn et al., 2008; Melkus et al., 2004; Speer et al., 2008), nurse educators (Nine et al., 2003; Gary et al., 2009; Melkus et al., 2004), diabetes educators (Quinn et al., 2011; Speer et al., 2008; Utz et al., 2008), curriculum-based approaches (Speer et al., 2008; Two Feathers et al., 2005; Magee et al., 2011), physician involvement (Quinn et al., 2008; Melkus et al., 2004; Utz et al., 2008), nutrition educators (Nine et al., 2003; Melkus et al., 2008; Speer et al., 2008), nurse case managers (Sekhobo et al., 2008), and use of telemedicine (Gary et al., 2009). Combinations of community intervention delivery methods were used in 12 of the 13 articles.

We found that the most common intervention delivery method that produced significant differences in HbA1C was group counseling (5 of 13 studies). Next was supervised exercise (4 studies), followed by nutritionist, community health worker, curriculum-based, and use of mobile device software (3 studies); culturally-tailored, nurse educator, physician involvement, one-on-one counseling, and diabetes educator (2 studies); and nurse case manager (1 study) (Table 3). Furthermore, we found that the only study that used telemedicine did not significantly reduce HbA1C.

Six of the studies described interventions whose study populations were only African Americans (Nine et al., 2003; Gary et al., 2009; Melkus et al., 2004; Utz et al., 2008; Magee et al., 2011; Murrock et al., 2009). There were 2 studies that included African Americans and Latinos (Spencer et al., 2011; Two Feathers et al., 2005). Three studies included Africans Americans and non-Hispanic Whites (Quinn et al., 2011; Quinn et al., 2008; Speer et al., 2008). Lastly, two of the 13 articles included African Americans, Hispanic Whites, and non-Hispanic Whites (Hargraves et al., 2012; Sekhobo et al., 2008). Information on each study’s sample population race/ethnicity is summarized in Table 1.

### 3.4 Efficacy of the Intervention

The level of evidence varied greatly across articles. Of the 5 randomized control trials, 3 reported improved glycemic control in the intervention group compared to the control group at the completion of the study. Of the 8 studies that were not randomized control trials, 6 showed a decrease in HbA1C that was statistically significant (p<0.05). Of the 13 studies only 2 indicated a follow-up or post intervention assessment, at 3 months or 6 months (Speer et al., 2008; Two Feathers et al., 2005).

## 4. Discussion

Thirteen articles met the inclusion criteria set for this review. The reviewed articles showed a variety of community intervention elements. In general, community interventions are shown to help manage T2DM in African Americans; however, no specific component is superior in achieving glycemic control. Overall, community interventions had a similar impact on glycemic control despite different intervention elements, ranging from 0.55% to 1.23% decrease in HbA1C, all being statistically significant (p<0.05) (Nine et al., 2003; Quinn et al., 2011; Quinn et al., 2008; Sekhobo et al., 2008; Two Feathers et al., 2005; Magee et al., 2011). A reduction in HbA1c greater than 0.5% is clinically significant and is associated with reduced risk of T_2_DM-related complications, specifically cardiovascular disease (Osende et al., 2001).

### 4.1 Implications of Community Interventions in African Americans

Our findings do not suggest that community health workers have a greater impact in attaining glycemic control compared to other community intervention elements. Rather, these findings suggest that several elements of community intervention studies that target African Americans with T2DM can have a positive effect on glycemic control. Our findings are in line with Norris and colleagues who indicated that diabetes self-management education, presented in a community setting, is an effective way to lower HbA1C and improve other T2DM related outcomes (Norris et al., 2002). Better glycemic control has been linked to improved T2DM outcomes, decreased complications, and improved quality of life (Tang et al., 2005). Therefore, the fact that community intervention studies are successful in reducing HbA1C is a significant finding.

Overall, this review provides evidence that community interventions are effective in African Americans with T2DM. However, the evidence shows that community interventions in the population of interest should not be heavily dependent on community health workers as the sole delivery mechanism to facilitate glycemic control. There are 3 elements of community interventions that were used in interventions that found statistically significant decreases in HbA1C: mobile device software, nutritionist educator, and curriculum-based approach (Table 3). Though no one study combined these 3 elements, future studies in this population should consider the use of these elements as part of community interventions. Additionally, future research should be conducted solely on African Americans to investigate the effectiveness of specific characteristics of community interventions in this population. Future studies may also find it important to use additional outcomes as a measure of effectiveness. For the purposes of replicating successful results, studies should provide more detailed descriptions of intervention techniques and measure the long-term impact on T2DM outcomes. Lastly, an intervention that includes a health system and community-based component may exponentially reduce HbA1C in African Americans.

### 4.2 Limitations

There are few noteworthy limitations of this systematic review. First, the review was limited to studies that were published in the English language between 2000 and 2012. Second, the review was limited to articles that had glycemic control as an outcome measure. There are other important diabetes outcomes that could be evaluated, such as risk factors associated with micro- and macrovascular disease. Third, publication bias and selective outcome reporting was possible and could have biased our conclusions. Fourth, there were various community intervention elements in the articles reviewed, which did not allow us to aggregate estimates of intervention effects or conduct a meta-analysis. Lastly, there are also limitations that can be derived from the 8 studies that were not RCTs, including increased inability to identify confounders, social desirability, and disproportionate number of participants in study groups.

In this review, risk of bias was likely present but no articles were excluded due to potential bias. The risk of bias could be introduced as a result of studies that did not show a statistically significant decrease in HbA1C due to small sample population, small number of potential study participants, inappropriate matching of control and intervention participants, researcher bias, and brief duration of the community intervention (Nine et al., 2003; Ueffing et al., 2012; Gary et al., 2009). Furthermore, studies with positive and statistically significant findings may be more likely to be published compared to studies who have negative or null findings.

## 5. Conclusion

The majority of the articles reviewed suggest that community interventions for T2DM self-management are effective in improving HbA1C in African Americans. Further research should be conducted where the study population is exclusively African Americans with T2DM so that the impact of various characteristics of community interventions on T2DM management can be assessed in this population. In the interim, the findings of this study provides insight for designing community-based interventions in the future, such as including use of multiple delivery methods, consideration of mobile device software, nutritionist educator, and curriculum-based approaches.
